# A 2004–2025 Bibliometric Study of Genetic Variation and Multiomics Biomarkers in Sepsis Based on 940 Publications

**DOI:** 10.1155/humu/7331886

**Published:** 2026-07-10

**Authors:** Qiuyan Yang, Siqi Yang, Yanyan Li, Dan Lv, Youbo Zuo

**Affiliations:** ^1^ Department of Emergency, Affiliated Hospital of North Sichuan Medical College, Nanchong, Sichuan, China, hospital-nsmc.com.cn; ^2^ Department of Anesthesiology, Affiliated Hospital of North Sichuan Medical College, Nanchong, Sichuan, China, hospital-nsmc.com.cn

**Keywords:** bibliometric analysis, biomarkers, genetic variation, multiomics, precision medicine, sepsis

## Abstract

Sepsis is a highly heterogeneous syndrome, and conventional clinical indicators and single biomarkers often fail to capture its biological complexity or support precise risk stratification. To clarify the development of research in this area, this bibliometric study analyzed the literature on the clinical translation of genetic variation and multiomics biomarkers in sepsis. Publications were retrieved from PubMed, Web of Science Core Collection, and Scopus for the period from 2004 to 2025, and 940 eligible records were analyzed using Bibliometrix, VOSviewer, and CiteSpace. The results showed rapid expansion of the field, with annual output increasing from 1 publication in 2004 to 185 in 2025. China and the United States emerged as the leading contributors, while major institutions and journals reflected strong interdisciplinary collaboration across critical care, molecular biology, and translational medicine. The knowledge structure of the field has evolved from early emphasis on polymorphisms, susceptibility, and conventional inflammatory biomarkers toward metabolomics, transcriptomics, genomics, precision medicine, machine learning, and Mendelian randomization. At the same time, clinically relevant themes such as mortality, septic shock, acute kidney injury, and neonatal sepsis have remained central. These findings map a research trajectory from exploratory biomarker discovery toward translational frameworks for sepsis endotyping, prognostic evaluation, and risk stratification, but they should not be interpreted as evidence that the identified biomarkers or models are already clinically implemented. Future progress will depend on multicenter prospective validation, standardized analytic pipelines, broader population representation, and clinically deployable models that can translate molecular heterogeneity into real‐world decision‐making.

## 1. Introduction

Sepsis is a life‐threatening organ dysfunction caused by a dysregulated host response to infection and remains one of the leading causes of death in critically ill patients worldwide [1–3]. The clinical management of patients with sepsis has significantly improved over the years with advancements in early recognition, antimicrobial therapy, organ support, and intensive care techniques [4, 5]. Conventional clinical criteria, inflammatory indices, and single biomarkers are the primary tools currently used for the diagnosis, risk stratification, and treatment monitoring of sepsis [6, 7]. These approaches are intended to facilitate timely intervention and improve patient outcomes, but an increasing number of studies have revealed that they do not sufficiently explain the marked biological heterogeneity of sepsis or accurately predict disease progression and prognosis [8–10]. However, once septic shock, persistent organ dysfunction, or severe secondary complications develop, the prognosis is often poor. Because sepsis is driven by complex interactions among infection, host immunity, inflammation, metabolism, coagulation, and organ injury, patients with similar clinical manifestations may still present with profoundly different molecular characteristics and clinical trajectories.

With the development of genomics, transcriptomics, proteomics, metabolomics, epigenomics, lipidomics, and other high‐throughput technologies, researchers have increasingly focused on variation, mutation, genetics, and multiomics biomarker signatures in sepsis [11, 12]. These approaches provide new opportunities to identify molecular endotypes and subphenotypes, improve early diagnosis, refine prognostic evaluation, monitor treatment response, and promote precision medicine in critically ill patients [13–15]. At the same time, a growing body of research has suggested that the clinical translation of sepsis biomarkers should move beyond isolated candidate molecules and toward multidimensional molecular frameworks that better reflect host–pathogen interactions and interpatient heterogeneity [16–18]. Nevertheless, the literature in this field is dispersed across critical care medicine, molecular biology, immunology, bioinformatics, and translational medicine, and there is still no universally accepted framework for converting omics‐derived variation and genetic findings into stable clinical tools [19, 20]. Therefore, gaining a comprehensive understanding of the research landscape, collaborative structure, intellectual basis, and emerging hotspots of this field is crucial for advancing individualized assessment and translational precision medicine in sepsis.

In this study, mutation‐ and variation‐oriented terms are treated together with multiomics biomarkers because they represent connected stages of the same translational continuum: inherited or acquired genetic variation may influence host susceptibility and immune response; multiomics layers capture the downstream transcriptional, protein, metabolic, epigenetic, lipid, and microbial consequences of this variation; and clinically oriented biomarker models attempt to convert these molecular signals into endotypes, prognostic tools, and treatment‐stratification frameworks. Therefore, “clinical translation” is defined here as progression from molecular discovery to reproducible endotyping, externally validated biomarkers or prediction models, and clinically deployable decision support, rather than simply the presence of an association in the literature.

**Figure 1:**
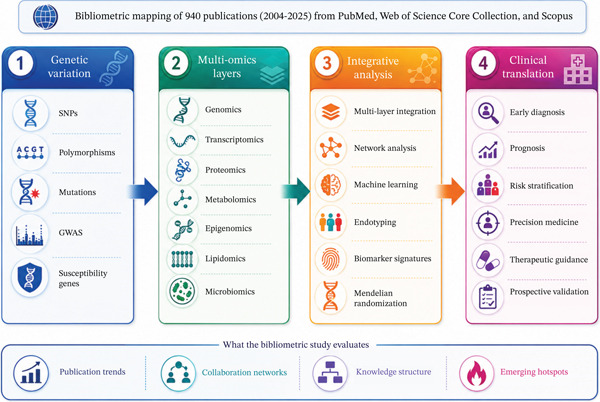
Conceptual and analytical framework of the study. The framework defines the study aim as a bibliometric mapping of the continuum from genetic variation (SNPs, polymorphisms, mutations, and GWAS) through multiomics integration (genomics, transcriptomics, proteomics, metabolomics, epigenomics, lipidomics, and microbiomics) to clinical translation, including endotyping, prognostic modeling, risk stratification, validation, and implementation.

Bibliometric analysis is an important method for systematically evaluating the knowledge structure and development trends of a scientific field. The objective of this study was to quantitatively map the evolution of research on genetic variation and multiomics biomarkers in sepsis and to evaluate, through publication trends, collaboration networks, citation structures, and keyword evolution, how the field has progressed along the continuum from genetic variation to multiomics integration and clinical translation (Figure 1).

## 2. Methods

### 2.1. Data Sources and Search Strategy

This bibliometric study was based on three complementary databases—PubMed, Web of Science Core Collection, and Scopus—to ensure broad coverage of clinical medicine, translational biomarker research, and omics‐oriented molecular investigation. The retrieval interval extended from January 1, 2004, to December 31, 2025. The final database retrieval and export for the revised dataset were performed on May 14, 2026; the publication‐year window was kept fixed at January 1, 2004, to December 31, 2025 to maintain a complete calendar‐year frame. No formal language restriction was applied during database retrieval, although the use of English search terms and English‐indexed metadata may still have introduced language‐related retrieval bias. The search strategy was structured around three conceptual domains: sepsis or septic shock; biomarker‐related constructs, including biomarkers, signatures, panels, endotypes, and subphenotypes; and multiomics or genetic dimensions, including genomics, transcriptomics, proteomics, metabolomics, epigenomics, lipidomics, microbiomics, mutation, variant, polymorphism, and related expressions of genetic variation. The formulation of search terms was adapted to the indexing logic of each database, but conceptual equivalence was maintained across platforms. Searches were conducted by using combinations of controlled vocabulary where available and free‐text terms in bibliographic fields such as title, abstract, author keywords, and indexed keywords. The initial strategy was intentionally sensitive rather than restrictive, so that clinically oriented studies, mechanistic translational studies, and biomarker development studies could be captured within a single retrieval framework. This approach was particularly important for the present topic, where relevant literature is dispersed across critical care, laboratory medicine, genomics, systems biology, and translational medicine journals. Records exported from the three databases were not analyzed separately. Instead, all retrieved citations were pooled after export to establish a unified source corpus. This design reduced the dependence of the final dataset on any single database and improved the likelihood that both mature and emerging areas of sepsis multiomics research were represented in the final analysis.

### 2.2. Eligibility Criteria and Literature Screening

Eligibility was defined a priori to retain records that addressed sepsis or septic shock as a primary disease context and examined biomarkers, omics‐derived signatures, molecular stratification, or genetic variation with direct or potential clinical relevance. Studies were considered eligible when they focused on diagnostic discrimination, early risk assessment, prognostic evaluation, patient stratification, endotyping, treatment‐response monitoring, or translational interpretation of multiomics and genetic findings in sepsis. The purpose of this definition was to distinguish clinically meaningful biomarker research from broader molecular work in infection or inflammation that did not specifically inform sepsis‐oriented clinical translation. Records were excluded when sepsis was incidental rather than central to the study question, when the work addressed nonseptic inflammatory disorders without a sepsis‐specific analytical frame, or when omics or genetic content was purely technical and unconnected to biomarker interpretation. Document types that do not provide stable bibliometric signals were removed before analysis, including editorials, letters, meeting abstracts, news items, notes, corrections, errata, book chapters, and duplicate early‐online versions. Duplicate removal was performed in a staged manner using DOI, PMID, normalized title strings, source information, first author, and publication year. When duplicate entries contained unequal metadata, the version with fuller affiliation and cited‐reference information was retained. A total of 4125 records were initially retrieved from PubMed, Web of Science Core Collection, and Scopus. After merging the records from the three databases and removing duplicates, 2852 unique records remained for screening. Literature screening was conducted sequentially by two team members, and disagreements or uncertain decisions at any stage were resolved through discussion by two senior investigators. First, title screening was performed to exclude clearly irrelevant records, leaving 2006 records for further assessment. Second, the abstracts of the remaining records were read in detail by the same two team members to evaluate whether the studies met the predefined eligibility criteria related to sepsis, genetic variation, multiomics biomarkers, molecular stratification, and clinical translational relevance. When disagreement occurred during abstract screening, the two senior investigators discussed the records and made the final inclusion decision. After abstract‐level assessment and metadata validation, 940 records were retained in the final analytical dataset. Screening was conducted sequentially. Titles and abstracts were first reviewed to remove clearly irrelevant material. Records that remained uncertain after abstract screening were assessed at the full bibliographic level, including indexed terms, author keywords, and cited‐reference fields. To minimize selection noise, bibliographic completeness was treated as part of eligibility for downstream mapping. After deduplication, relevance screening, and metadata validation, 940 records were retained in the final analytical dataset.

### 2.3. Data Conversion and Bibliographic Standardization

Because PubMed, Web of Science, and Scopus differ substantially in export architecture and field tagging, all retained records were converted into a unified Web of Science–compatible format before analysis. This harmonization step was necessary to ensure that the three software environments operated on the same bibliographic structure and that discrepancies in tags did not generate artificial differences in node counts, citation links, or collaboration networks. The converted corpus preserved the principal fields required for science mapping, including title, authors, publication year, source title, abstract, author keywords, indexed keywords where available, affiliations, country information, cited references (CR), and persistent identifiers. Standardization was then performed at several levels. Author names were normalized to reduce fragmentation caused by variations in initials, spacing, hyphenation, or surname order. Institutional names were reconciled across abbreviations, affiliated hospitals, and alternative English renderings of the same parent organization. Country names were unified to a single contemporary form. Journal titles were standardized across abbreviated and full‐title variants. Keyword cleaning was particularly important for this field because conceptually identical terms frequently appear in multiple forms. Singular and plural variants, hyphenated and nonhyphenated forms, spelling differences, and common synonym pairs were merged after review. Terms such as multi‐omics and multiomics, or SNP and single‐nucleotide polymorphism, were normalized only when semantic equivalence was clear. A thesaurus‐based cleaning procedure was first applied, followed by manual verification of high‐frequency entities to avoid inappropriate aggregation of biologically distinct concepts. Records with incomplete, conflicting, or evidently corrupted metadata were traced back to the original export files and corrected where possible. Only after this standardization process was completed, the final dataset was submitted to bibliometric analysis. The cleaned Web of Science–format corpus served as the common input for CiteSpace, VOSviewer, and Bibliometrix throughout the study.

### 2.4. Bibliometric Mapping and Knowledge‐Structure Analysis

The analytical framework combined descriptive bibliometrics with network‐based science mapping in order to characterize productivity, collaboration, intellectual structure, and thematic evolution within the field. Bibliometrix in R was used for the general descriptive profile of the literature, including annual scientific production, annual growth, source productivity, citation summaries, author‐level output, country participation, corresponding‐author country distribution, and cross‐field linkages among authors, institutions, and countries. These analyses established the macrostructure of the dataset and provided the baseline indicators required for subsequent network interpretation. VOSviewer was used to construct and visualize relational networks because of its stability in dense bibliographic datasets and its suitability for coauthorship, cocitation, and keyword co‐occurrence mapping. Coauthorship networks were generated for authors, institutions, and countries to examine collaborative structure and leadership patterns. Cocitation analysis was used to identify the intellectual backbone of the field at the levels of references and journals. Keyword co‐occurrence analysis was used to delineate the conceptual architecture of sepsis biomarker research across multiomics and genetic themes. In these maps, node size represented frequency or productivity, link width reflected the strength of association, and total link strength was used as the principal index of relational intensity. CiteSpace was used for time‐sensitive and structurally diagnostic analyses, including keyword timeline mapping, burst detection for keywords and references, journal dual‐map overlay, and identification of nodes with high intermediary importance. The publication range was sliced by year to capture thematic emergence, persistence, and transition across the study period. Thresholds for network display were determined iteratively according to node frequency and map density rather than imposed mechanically, so that the resulting visualizations preserved the dominant structure of the field without excessive fragmentation. Together, these procedures enabled simultaneous examination of research growth, collaborative organization, citation influence, and the evolution of major knowledge domains.

For figure interpretation, each caption now specifies the meaning of node size, link width, color gradient, and special rings. In CiteSpace maps, pink or purple outer rings indicate nodes with higher betweenness centrality and therefore a potential bridging role in the network; in VOSviewer overlay maps, the color gradient represents the average publication year of the node, whereas larger nodes and thicker links indicate greater productivity/frequency and stronger relationships, respectively.

### 2.5. Statistical Methods

**Figure 2:**
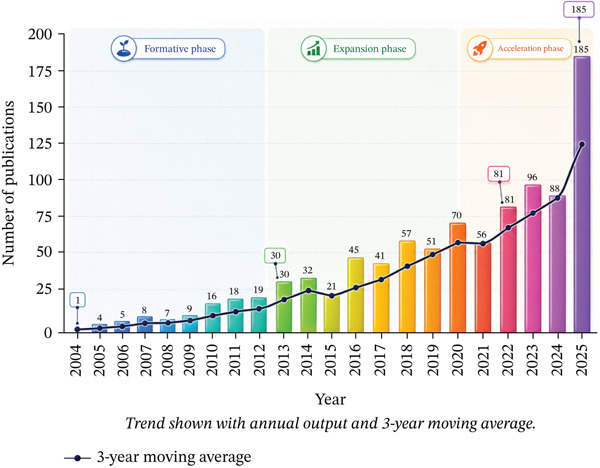
Annual publication output and growth trend in sepsis genetic variation and multiomics biomarker research, 2004–2025. Bars indicate the annual number of included publications. The line indicates the 3‐year moving average, which was added to reduce overinterpretation of unstable year‐to‐year percentage changes in early years with very small counts. Year‐to‐year growth rates are provided in Table S1.

This study used a descriptive bibliometric design. The purpose was to characterize the structure and development of a research field rather than to test a clinical hypothesis; accordingly, the analysis emphasized bibliographic indicators, network measures, and temporal patterns instead of inferential comparison. Temporal productivity was evaluated using the annual number of publications, the proportion of total output contributed by each year, and annual growth rate. The growth rate was defined as the relative change in publication count compared with the immediately preceding year. Citation impact was assessed using total citations, average citations per document, and h‐index at the author, journal, institution, and country levels where appropriate. Because citation counts are strongly affected by publication age, citation‐based metrics were interpreted alongside productivity measures rather than in isolation. Recent records were therefore assessed primarily for emerging thematic relevance and network position, whereas older records contributed more strongly to cocitation structure and cumulative influence. Collaboration was evaluated through the number of participating countries and institutions, corresponding‐author country leadership, and network connectivity. In VOSviewer, total link strength was used to quantify the intensity of collaboration or cocitation. In CiteSpace, betweenness centrality was used to identify nodes occupying structurally bridging positions in the network. For thematic analysis, keyword frequency, co‐occurrence strength, cluster membership, timeline persistence, and burst intensity were used jointly to identify established themes and emerging fronts. When clustering results were generated in CiteSpace, their structural reliability was appraised using modularity and silhouette statistics, when applicable. VOSviewer clusters were interpreted according to network density and semantic coherence. All analyses were performed on the fully standardized 940‐record corpus after completion of deduplication and metadata cleaning. To enhance internal consistency, the principal descriptive patterns and leading nodes were cross‐checked across Bibliometrix, VOSviewer, and CiteSpace before final interpretation. For annual growth analysis, the first year was recorded as “not applicable” because no previous‐year denominator existed. Because very large percentage changes can be generated by small early publication counts, the revised Figure 2 emphasizes annual output and a three‐year moving average, whereas year‐to‐year growth rates in Table S1 are interpreted descriptively and cautiously.

## 3. Results

### 3.1. Analysis of Publication Volume Trends and Overall Status of the Field

From 2004 to 2025, publications on genetic variation and multiomics biomarkers in sepsis increased from 1 article in 2004 to 185 articles in 2025 (Figure 2 and Table S1). Output remained low during 2004–2012, generally below 20 publications per year, increased after 2013, and exceeded 80 publications per year from 2022 onward. Because the early annual counts were small, the large year‐to‐year percentage changes in the first decade should be interpreted as descriptive signals rather than stable growth estimates. The revised trend plot therefore reports the annual number of publications together with a 3‐year moving average. The 2025 count was retained because the search window ended on December 31, 2025, and the final export was performed after that date; however, the value should be interpreted as complete only relative to the database indexing status on the retrieval date. The general bibliometric profile showed 940 documents from 408 sources, 6584 authors, 20 single‐authored papers, 8.76 coauthors per document, and an international coauthorship rate of 25.64% (Figure S1). The three‐field plot linked CR, authors (AU), and merged keywords (KW_Merged), indicating that foundational references on sepsis definitions, epidemiology, and biomarker discovery were connected with high‐frequency terms such as sepsis, biomarkers, septic shock, metabolomics, mortality, proteomics, and transcriptomics (Figure S2).

### 3.2. Country Analysis

**Figure 3:**
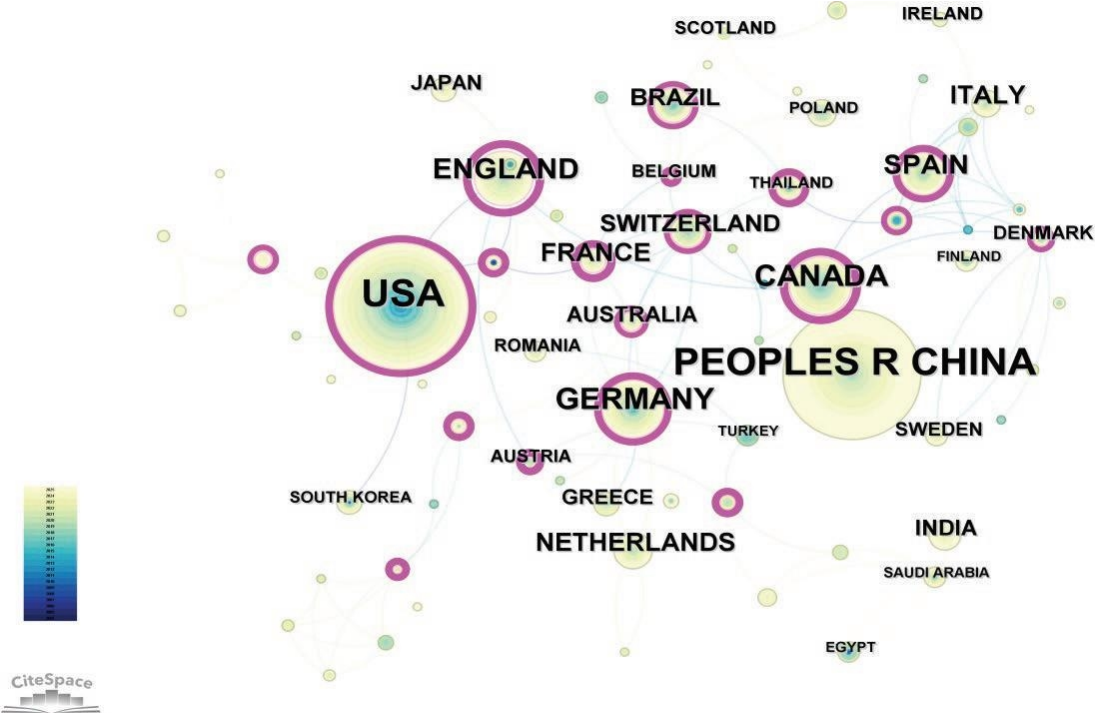
Country collaboration network in sepsis genetic variation and multiomics biomarker research. Each node represents a country, node size reflects publication frequency, and links represent coauthorship collaboration. Pink or purple outer rings in the CiteSpace map denote nodes with higher betweenness centrality, indicating potential bridging roles between country clusters rather than a separate country category.

The country‐level distribution showed a globally distributed but concentrated research landscape. In the CiteSpace country collaboration map, the network comprised 78 nodes and 111 links, with a density of 0.037 (Figure 3). China (People′s Republic of China) and the United States were the two largest publication contributors, followed by Canada, Germany, the United Kingdom, Italy, Spain, France, the Netherlands, and Switzerland. The VOSviewer overlay map further showed temporal variation in country participation, with earlier average publication years in several North American and Western European nodes and more recent activity in China and selected Asian settings (Figure S3). The corresponding‐author country bar chart provides the direct data support for corresponding‐author leadership: China ranked first, the United States ranked second, and both countries contributed mainly single‐country publications while also maintaining multicountry publications (Figure S4). Because these patterns are descriptive bibliometric indicators, causal explanations for national output differences are discussed only in Section 4.

### 3.3. Analysis of Research Institutions

The institutional collaboration network showed a concentrated but broadly connected research structure. In the VOSviewer overlay map, the network was centered on institutions such as the University of Pennsylvania, University of British Columbia, Shanghai Jiao Tong University, University of Calgary, Stanford University, University of California San Francisco, and Johns Hopkins University, which showed relatively strong links with other institutional nodes (Figure S5). The overlay visualization also showed temporal differences in institutional participation, with earlier average publication years mainly observed in several North American and European institutions and more recent average publication years appearing in Chinese institutions and other emerging collaborators.

CiteSpace provided a complementary institutional network containing 198 nodes and 363 links, with a network density of 0.0093, indicating a low‐density but connected collaboration structure (Figure S6). In this map, the University of California System, Harvard University Medical Affiliates, University System of Ohio, Imperial College London, and several European research consortia occupied relatively prominent or bridging positions. The productivity ranking further showed that the University of California System ranked first with 110 publications, followed by Harvard University (87), University of Calgary (81), and University of Amsterdam (72). The University of Michigan, University of Michigan System, Pennsylvania Commonwealth System of Higher Education, Friedrich Schiller University of Jena, University System of Ohio, and Western University also contributed substantial publication output (Figure S7). The institutional results showed that research on genetic variation and multiomics biomarkers in sepsis was concentrated in several high‐output university systems and academic medical centers, whereas collaboration links extended across a wider set of participating institutions.

### 3.4. Journal Analysis

The journal structure of this field shows a clear separation between the sources in which studies are most frequently published and the sources that provide the main intellectual foundation. The journal co‐occurrence map reveals three tightly connected but distinguishable clusters: (1) centered on immunology and molecular medicine journals such as Frontiers in Immunology, International Journal of Molecular Sciences, and Scientific Reports; (2) centered on clinical critical care and respiratory journals such as Critical Care, American Journal of Physiology, Critical Care Clinics, and Lancet Respiratory Medicine; and (3) linked to proteomics, metabolomics, and analytical bioscience journals, including RSC Advances, Metabolites, Journal of Proteomics, and related sources (Figure S8). The local citation structure further clarifies the hierarchy of influence. Critical Care Medicine (1735 local citations) and Critical Care (1390) dominate the cited‐source ranking, followed by PLOS ONE (1117), American Journal of Respiratory and Critical Care Medicine (1078), JAMA (843), Intensive Care Medicine (826), and New England Journal of Medicine (806), indicating that the field continues to rely heavily on journals that define sepsis, establish clinical endpoints, and frame the interpretation of biomarkers in relation to diagnosis, organ dysfunction, and prognosis (Figure S9). By contrast, the most productive publication venues are led by Scientific Reports (39 articles), Frontiers in Immunology (34), Critical Care (26), PLOS ONE (24), and International Journal of Molecular Sciences (23), with additional contributions from American Journal of Respiratory and Critical Care Medicine, RSC Advances, Journal of Proteome Research, Critical Care Medicine, and Metabolites (Figure S10). These descriptive results show a stable clinical citation core and a diversified publication periphery.

### 3.5. Keyword Analysis

**Figure 4:**
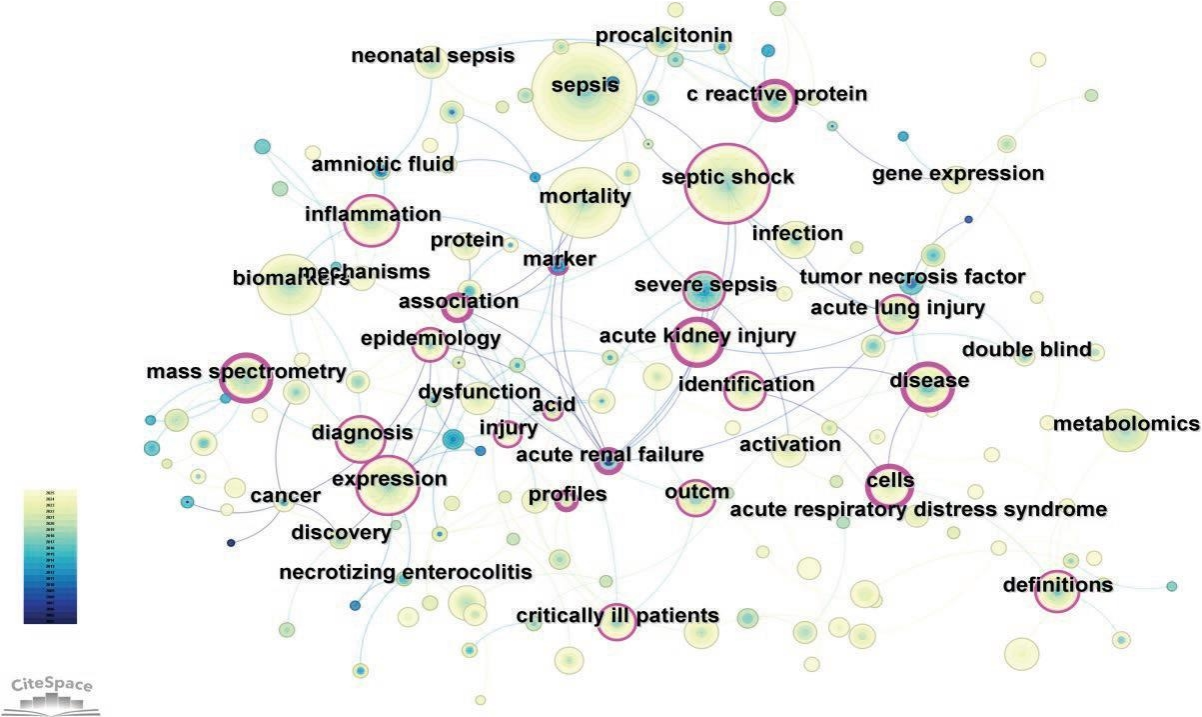
Keyword co‐occurrence network in sepsis genetic variation and multiomics biomarker research. Each node represents a keyword, node size reflects keyword frequency, links represent co‐occurrence strength, and pink or purple rings in the CiteSpace map highlight keywords with higher betweenness centrality. The map supports interpretation of four thematic categories: genetic variation, omics technologies, immune dysregulation, and clinical translation.

The keyword structure was reorganized into four thematic categories to clarify the conceptual architecture of the field. First, the genetic‐variation category included terms such as polymorphism, polymorphisms, susceptibility, gene expression, mutation‐related terminology, and genomic profiling, reflecting early candidate‐gene and variant‐association work and its later extension into broader genomic analysis. Second, the omics‐technology category included transcriptomics, proteomics, metabolomics, genomics, lipidomics, mass spectrometry, profiles, and biomarker discovery, indicating the increasing use of high‐throughput molecular layers. Third, the immune‐dysregulation category included inflammation, tumor necrosis factor, cytokine‐related terms, infection, immune response, and organ‐dysfunction terms such as acute kidney injury, showing that molecular biomarker research remained anchored in host‐response biology. Fourth, the clinical‐translation category included biomarkers, diagnosis, prognosis, mortality, septic shock, neonatal sepsis, precision medicine, machine learning, and Mendelian randomization, highlighting the movement from discovery toward stratification, prediction, and causal interpretation. In the CiteSpace co‐occurrence network, the keyword map contained 230 nodes and 351 links, with sepsis, septic shock, mortality, biomarkers, infection, gene expression, metabolomics, acute kidney injury, procalcitonin, and C‐reactive protein among the largest or most connected terms (Figure 4). The VOSviewer overlay map showed a temporal shift from earlier attention to polymorphism, susceptibility, definitions, and conventional inflammatory markers toward more recent emphasis on metabolomics, transcriptomics, genomics, biomarker discovery, precision medicine, machine learning, lipidomics, and COVID‐19 (Figure S11). Cluster and timeline analyses further supported a staged trajectory in which clinical problems such as procalcitonin, acute kidney injury, and neonatal sepsis persisted, whereas newer clusters such as precision medicine, machine learning, and Mendelian randomization became more visible (Figures S12 and S13).

## 4. Discussion

Sepsis remains one of the most complex and heterogeneous syndromes in critical care, and despite continuous advances in supportive treatment, early diagnosis, prognostic assessment, and treatment stratification are still difficult in a considerable proportion of patients [21, 22]. Conventional clinical criteria and routine inflammatory indicators are useful for syndromic recognition, but they are often insufficient to fully reflect the profound biological heterogeneity that underlies differences in host response, organ dysfunction, and outcome [23, 24]. In recent years, this limitation has driven increasing interest in biomarkers derived from genomics, transcriptomics, proteomics, metabolomics, epigenetics, and other multiomics frameworks, as well as in genetic variation and mutation‐related research, with the aim of refining sepsis phenotypes and improving clinical translation [25, 26]. However, although this literature has expanded rapidly, its overall developmental trajectory, knowledge structure, and translational orientation have not been systematically clarified. Therefore, we performed a bibliometric study to characterize the evolution of research on the clinical translation of genetic variation and multiomics biomarkers in sepsis and to identify the major collaborative hubs, intellectual foundations, and emerging thematic frontiers in this field. Throughout this discussion, “translation” is used cautiously to mean movement toward clinically testable endotypes, biomarkers, and predictive models, not proof of current bedside readiness.

The publication trend should be interpreted as evidence of a rapidly expanding research agenda, not as direct evidence of clinical maturity. The early stage of the field was dominated by candidate‐gene, polymorphism, and single‐marker studies, which were useful for identifying susceptibility hypotheses but were often limited by small cohorts, inconsistent sepsis definitions, platform heterogeneity, and insufficient external validation [27, 28]. The later rise of transcriptomic, proteomic, metabolomic, and computational approaches reflects a shift from isolated marker discovery toward systems‐level endotyping and biomarker panels [29, 30]. This interpretation is consistent with previous bibliometric work showing that sepsis omics research has expanded unevenly across regions and technologies, and with broader sepsis bibliometric studies showing sustained growth of sepsis publications while also identifying specialization into subfields such as immunosuppression and omics‐oriented biomarker research [31–33].

Practically, multiomics integration in this field appears to occur through three main routes. The first is multilayer integration, in which genomics or genetic variation is analyzed together with transcriptomic, proteomic, metabolomic, epigenomic, lipidomic, or microbiome data from the same or related patient populations. The second is network‐based integration, in which correlated genes, proteins, metabolites, pathways, and clinical endpoints are organized into modules or interaction networks. The third is machine‐learning integration, in which high‐dimensional molecular features are combined with clinical variables to support diagnosis, prognosis, endotype assignment, or risk stratification [19, 20, 34, 35]. These approaches can improve biological interpretation because they connect relatively stable host variation with dynamic immune, metabolic, and organ‐injury responses; nevertheless, they also increase the need for harmonized sampling, transparent modeling, and external validation.

The geographic distribution of publications further indicates that this field has developed as a globally connected but unequally concentrated research domain. China and the United States occupy the two leading positions in productivity and visibility, whereas Canada, Germany, the United Kingdom, Italy, Spain, France, the Netherlands, and Switzerland form a second tier of active contributors. This pattern is plausible because multiomics biomarker translation in sepsis requires access to large patient populations, standardized biospecimen collection, intensive care networks, sequencing platforms, computational expertise, and sustained financial support, all of which are more available in large and mature biomedical research systems [36, 37]. The recent rise of China and several Asian contributors also suggests that the geographic center of activity is broadening beyond its earlier Euro‐American core. However, it is equally important to note that regions bearing a substantial sepsis burden remain underrepresented. This imbalance has important translational implications. Biomarkers, endotypes, and prediction models developed primarily in high‐income or upper–middle‐income settings may not generalize well to populations with different pathogen distributions, host genetic backgrounds, comorbidity structures, or resource constraints [38, 39]. Therefore, although the existing leadership structure has accelerated scientific development, broader international inclusion will be necessary if the field is to move from high‐volume discovery toward truly global clinical applicability.

The institutional and journal landscapes provide a complementary perspective on how the field is organized. The concentration of institutional output in large university systems and academic medical centers may reflect the resource‐intensive nature of sepsis genetic‐variation and multiomics research. Such studies usually require access to well‐characterized intensive care cohorts, standardized biospecimen collection, sequencing or multiomics platforms, bioinformatics infrastructure, and clinically interpretable endpoints. Therefore, leadership in this field is likely shaped not only by publication productivity but also by the ability to integrate critical care medicine, molecular profiling, computational analysis, and multicenter collaboration within durable organizational platforms. The temporal pattern observed in the institutional overlay map further suggests that the institutional base of the field is gradually broadening, with more recent activity appearing in Chinese institutions and other emerging collaborators as omics technologies and hospital‐based translational programs become more widely available.

At the journal level, a clear distinction appears between the journals that publish a large number of studies and the journals that provide the main citation backbone of the field. On the one hand, productive sources include broad‐scope and open‐access journals that are well suited to exploratory and data‐intensive omics research. On the other hand, the most influential cited journals remain anchored in critical care medicine, respiratory medicine, and high‐impact general medicine. This suggests that even when biomarker discovery is methodologically dispersed across molecular and analytical venues, its clinical legitimacy continues to depend on the core literature that defines sepsis, clarifies organ dysfunction, and frames clinically meaningful outcomes [40, 41]. In other words, the field is methodologically interdisciplinary but clinically anchored, and this dual structure is one of its defining characteristics.

The thematic evolution revealed by the keyword analyses is perhaps the most important signal of how the field is changing. Earlier work was more closely associated with polymorphisms, susceptibility, disease definitions, and conventional inflammatory markers, whereas recent research increasingly emphasizes metabolomics, transcriptomics, genomics, biomarker discovery, precision medicine, machine learning, and, to a lesser extent, lipidomics and COVID‐19. This pattern indicates a shift from candidate‐marker logic toward systems‐level biological characterization. In the earlier phase, the field largely asked whether a particular marker or genetic variant could distinguish sepsis or predict severity. In the more recent phase, the field is increasingly asking whether integrated omics signatures can define endotypes, reveal clinically meaningful biological subgroups, and improve prognostic or therapeutic stratification [42, 43]. This transition is highly consistent with the broader evolution of sepsis research. Because sepsis is not a single biological entity but a syndrome arising from highly variable host–pathogen interactions, it is increasingly unlikely that a single universal biomarker will be sufficient for diagnosis, prognosis, and therapeutic decision‐making across all patients. The current frontier, therefore, is less about identifying one dominant molecule and more about building coherent molecular frameworks that can capture heterogeneity in a clinically interpretable way.

At the same time, our results also show that the field has not abandoned stable bedside problems. High‐frequency terms such as mortality, acute kidney injury, procalcitonin, C‐reactive protein, infection, and septic shock remain central, and several of the major clusters continue to revolve around specific clinical contexts such as neonatal sepsis and organ dysfunction. This is an important observation. It suggests that the literature is not moving away from clinical utility toward purely technical complexity; rather, it is attempting to connect new omics methodologies to longstanding practical problems in sepsis care. The persistent visibility of procalcitonin and C‐reactive protein indicates that conventional biomarkers remain embedded in the translational conversation, not because they fully solve the problem, but because they continue to serve as clinical reference points against which newer molecular approaches are evaluated [44, 45]. Likewise, the continued prominence of acute kidney injury and neonatal sepsis reflects areas in which diagnostic uncertainty, biological vulnerability, and prognostic urgency are especially high, making biomarker‐guided decision‐making particularly attractive [46, 47]. This may suggest that the most successful translational progress in the near term will emerge not from attempting to replace all conventional markers at once, but from combining established clinical indicators with multiomics features in targeted high‐need scenarios.

Another notable finding is the increasing appearance of machine learning, precision medicine, and Mendelian randomization within the recent thematic structure. Their rising visibility is meaningful because each addresses a different limitation of earlier biomarker research. Machine learning reflects an attempt to handle high‐dimensional and multimodal data more effectively and to translate complex patterns into predictive tools. Precision medicine reflects the growing ambition to move beyond uniform treatment frameworks and toward subgroup‐based clinical management. Mendelian randomization reflects an effort to strengthen causal inference and reduce the interpretive limitations of purely associative biomarker studies [48, 49]. Together, these themes suggest that the field is entering a more analytically mature phase. However, they also reveal the central challenge of clinical translation. Sophisticated analytical models and complex omics signatures can improve biological resolution, but they do not automatically guarantee bedside utility. For real translation to occur, biomarkers must not only show statistical association but also demonstrate reproducibility across cohorts, robustness across platforms, compatibility with clinically relevant time windows, and clear incremental value over existing tools. Therefore, the rise of these newer approaches should be interpreted not as evidence that the translational problem has been solved, but as evidence that the field is actively trying to overcome the limitations of earlier descriptive work.

The emerging translational hotspots can be summarized as multiomics‐based endotyping, machine‐learning–driven predictive models, integration of genetic variation with downstream omics signatures, biomarker‐guided risk stratification in septic shock and organ dysfunction, and precision sepsis phenotyping for trial enrichment. These areas show increasing translational orientation because they attempt to connect molecular heterogeneity with interpretable clinical endpoints. However, bibliometric prominence should not be interpreted as evidence of clinical adoption. The literature still remains closer to translational development than routine implementation, and the most important next step is prospective, multicenter testing against clinically meaningful decision points.

From a translational perspective, our findings indicate that the main bottleneck is now shifting from discovery to validation and implementation. The rapid growth of omics‐based studies shows that the field has generated a large number of candidate signals and promising molecular classifications. However, the coexistence of discovery‐oriented themes with relatively fewer implementation‐ and validation‐oriented signals suggests that the field remains closer to translational development than routine clinical deployment. This interpretation should be regarded as bibliometric inference rather than direct evidence of validation status. This is a familiar pattern in biomarker science. Sepsis studies are particularly vulnerable to heterogeneity in sampling time, disease definition, pathogen spectrum, treatment exposure, comorbidity burden, and analytic workflow, all of which can affect reproducibility and limit generalizability [50, 51]. Integrating genetic variation and multiomics data into clinically actionable frameworks requires harmonized biospecimen procedures, standardized preprocessing pipelines, transparent modeling strategies, and prospective testing in appropriately annotated patient cohorts. Without these steps, the field risks producing increasingly sophisticated molecular associations without achieving commensurate clinical adoption. Therefore, the future value of this research area will likely depend less on expanding the number of candidate biomarkers and more on improving standardization, validation, and interpretability. As shown in Table 1, to make this trajectory explicit, the revised manuscript summarizes the field in a stage‐based translational table that separates foundational genetic‐variation studies, multiomics integration studies, clinical‐problem biomarker studies, and implementation‐oriented validation efforts.

**Table 1:** Stage‐based translational trajectory of genetic variation and multiomics biomarkers in sepsis.

Stage	Bibliometric signals	Meaning for translation	Main gap
Early genetic‐variation discovery	SNPs; polymorphisms; mutations; susceptibility; gene expression; GWAS	Generated host‐susceptibility hypotheses and linked variation to immune response.	Small cohorts and inconsistent replication.
Expansion into multiomics integration	Genomics; transcriptomics; proteomics; metabolomics; epigenomics; lipidomics; microbiomics	Connects molecular layers to pathways, modules, and endotypes.	Requires standardized sampling, platforms, and interpretable integration.
Clinical‐problem anchoring	Mortality; septic shock; acute kidney injury; neonatal sepsis; procalcitonin; C‐reactive protein	Anchors omics signals to endpoints and high‐need patient groups.	Prospective incremental value over existing tools remains unproven.
Implementation‐oriented validation	Precision medicine; machine learning; Mendelian randomization; validation	Supports endotype‐guided prediction, trial enrichment, and decision support.	Needs external validation, real‐time feasibility, and proof of patient benefit.

*Note:* The stages are intended to summarize the dominant translational logic of the field rather than to imply that all studies followed a strictly linear sequence.

### 4.1. Limitations

Although this study provides a comprehensive overview of the field, several limitations need to be acknowledged. First, bibliometric analyses are influenced by database coverage, indexing logic, export structure, and metadata quality. Although PubMed, Web of Science Core Collection, and Scopus were integrated, relevant studies may still have been missed or differently represented. Second, the 2025 output should be interpreted as complete only according to the indexing status of the databases on the final retrieval date; subsequent indexing updates could change the exact count. Third, no formal language restriction was applied, but the use of English search terms and English‐indexed metadata may have underrepresented non‐English literature, which could influence country and institution analyses. Fourth, annual growth rates in the early period should be interpreted cautiously because very small denominators can produce unstable percentage changes. Fifth, citation‐based indicators favor older publications, whereas recent studies may be thematically important but undercited. Sixth, keyword cleaning and entity normalization are necessary for science mapping but can merge semantically related terms or preserve distinctions that later prove biologically important. Seventh, network figures remain inherently dense; although captions were expanded and the three‐field plot was moved to the supporting information, fine labels in complex maps should be interpreted together with the textual results rather than as standalone evidence. Finally, bibliometric prominence cannot establish methodological quality, patient‐level benefit, regulatory readiness, or routine clinical implementation. Because the present study maps literature structure rather than extracting trial‐level validation outcomes, it cannot quantify the exact proportion of top‐cited papers that progressed to clinical adoption. This unresolved translational question should be addressed in future systematic reviews or evidence‐mapping studies focused specifically on validation status and clinical utility.

The present study shows that research on genetic variation and multiomics biomarkers in sepsis has entered a more organized and translationally ambitious stage. The field is moving away from a narrow search for single universal markers and toward a broader effort to define biologically meaningful subphenotypes, integrate multidimensional molecular data, and support more precise clinical stratification. This trend is promising, but the bibliometric evidence should be read as a map of scientific development rather than proof of clinical readiness. The next phase will require multicenter prospective cohorts, broader geographic and ethnic representation, reproducible analytic pipelines, explicit reporting of model performance across cohorts, and closer integration between omics‐derived signatures and clinically interpretable endpoints. Only through such efforts can the promise of genetic variation and multiomics biomarkers in sepsis move from a rapidly expanding research frontier toward routine clinical value.

## 5. Conclusion

In this bibliometric study of 940 publications from 2004 to 2025, the literature on genetic variation and multiomics biomarkers in sepsis was mapped as a rapidly expanding and increasingly structured research field. The results show growth in annual output, leading contributions from China and the United States, and a thematic transition from early polymorphism and conventional inflammatory‐marker studies toward metabolomics, transcriptomics, genomics, precision medicine, machine learning, and Mendelian randomization. These findings suggest future priorities for sepsis research, including multiomics‐based endotyping, biomarker‐guided risk stratification, AI‐assisted predictive modeling, and cross‐population validation. Importantly, the study does not demonstrate that these biomarkers or models are ready for routine clinical implementation. Progress toward clinical translation will require standardized biospecimen workflows, transparent analytic pipelines, multicenter prospective validation, broader population representation, and clinically deployable models linked to real diagnostic, prognostic, and treatment‐stratification decisions.

## Author Contributions

Conceptualization: Qiuyan Yang, Siqi Yang, and Youbo Zuo. Methodology: Qiuyan Yang, Siqi Yang, Yanyan Li, and Youbo Zuo. Validation: Siqi Yang, Yanyan Li, Dan Lv, and Youbo Zuo. Formal analysis: Qiuyan Yang, Siqi Yang, and Youbo Zuo. Investigation: Qiuyan Yang, Siqi Yang, Dan Lv, and Youbo Zuo. Resources: Qiuyan Yang, Siqi Yang, Yanyan Li, and Dan Lv. Data curation: Qiuyan Yang, Siqi Yang, Yanyan Li, and Dan Lv. Writing—original draft: Qiuyan Yang, Siqi Yang, and Yanyan Li. Writing—review & editing: Qiuyan Yang, Siqi Yang, and Youbo Zuo. Project administration: Qiuyan Yang and Siqi Yang. Qiuyan Yang and Siqi Yang contributed equally to this work.

## Funding

This work was supported by the Primary Health Development Research Center of Sichuan Province Program (SWFZ23‐Y‐53).

## Disclosure

All authors have read and agreed to the published version of the manuscript.

## Ethics Statement

This study was reviewed by the Ethics Committee of the Affiliated Hospital of North Sichuan Medical College. Following the review, the ethics committee determined that this study could proceed without requiring approval from the ethics committee.

## Conflicts of Interest

The authors declare no conflicts of interest.

## Supporting information


**Supporting Information** Additional supporting information can be found online in the Supporting Information section. The following supporting files are available online. Table S1 provides annual publication counts, percentage of total output, and year‐on‐year growth rates for the period of 2004–2025. Figures S1, S2, S3, S4, S5, S6, S7, S8, S9, S10, S11, S12, and S13 include the following: the general bibliometric profile of the 940‐record corpus (Figure S1); the three‐field plot of cited references, authors, and merged keywords (Figure S2); overlay visualizations of international collaboration and corresponding‐author country distribution (Figures S3 and S4); institutional collaboration overlay and CiteSpace network maps with productivity rankings (Figures S5, S6, and S7); journal source network and citation and productivity rankings (Figures S8, S9, and S10); and keyword evolution overlay map, clustering map, and timeline visualization (Figures S11, S12, and S13).

## Data Availability

The bibliographic data analyzed in this study were retrieved from PubMed, Web of Science Core Collection, and Scopus. Restrictions apply to the availability of the Web of Science Core Collection and Scopus data, which were accessed under institutional license for this study. The standardized bibliometric dataset generated during the current study is available from the corresponding author upon reasonable request and with the permission of the respective database providers.
