# Supplementary material for: A 2004–2025 Bibliometric Study of Genetic Variation and Multiomics Biomarkers in Sepsis Based on 940 Publications
Source: Hum Mutat. 2026 Jul 10;2026:7331886. doi: 10.1155/humu/7331886 (PMC13352517; doi:10.1155/humu/7331886)
Supplement: Supplementary file 1 — Supporting Information Additional supporting information can be found online in the Supporting Information section. The following supporting files are available online. Table S1 provides annual publication counts, percentage of total output, and year‐on‐year growth rates for the period of 2004–2025. Figures S1, S2, S3, S4, S5, S6, S7, S8, S9, S10, S11, S12, and S13 include the following: the general bibliometric profile of the 940‐record corpus (Figure S1); the three‐field plot of cited references, authors, and merged keywords (Figure S2); overlay visualizations of international collaboration and corresponding‐author country distribution (Figures S3 and S4); institutional collaboration overlay and CiteSpace network maps with productivity rankings (Figures S5, S6, and S7); journal source network and citation and productivity rankings (Figures S8, S9, and S10); and keyword evolution overlay map, clustering map, and timeline visualization (Figures S11, S12, and S13). [file HUMU-2026-7331886-s001.docx]

**Supplementary Information**

**Supplementary Table 1. Number of publications and growth rate over the years.**

| **Year** | **Articles** | **Percentage of the total** | **Growth rate** |
| --- | --- | --- | --- |
| 2004 | 1 | 0.106% | Not applicable |
| 2005 | 4 | 0.426% | 300.000% |
| 2006 | 5 | 0.532% | 25.000% |
| 2007 | 8 | 0.851% | 60.000% |
| 2008 | 7 | 0.745% | -12.500% |
| 2009 | 9 | 0.957% | 28.571% |
| 2010 | 16 | 1.702% | 77.778% |
| 2011 | 18 | 1.915% | 12.500% |
| 2012 | 19 | 2.021% | 5.556% |
| 2013 | 30 | 3.191% | 57.895% |
| 2014 | 32 | 3.404% | 6.667% |
| 2015 | 21 | 2.234% | -34.375% |
| 2016 | 45 | 4.787% | 114.286% |
| 2017 | 41 | 4.362% | -8.889% |
| 2018 | 57 | 6.064% | 39.024% |
| 2019 | 51 | 5.426% | -10.526% |
| 2020 | 70 | 7.447% | 37.255% |
| 2021 | 56 | 5.957% | -20.000% |
| 2022 | 81 | 8.617% | 44.643% |
| 2023 | 96 | 10.213% | 18.519% |
| 2024 | 88 | 9.362% | -8.333% |
| 2025 | 185 | 19.681% | 110.227% |

Note. Growth rate for 2004 is not applicable because no previous-year denominator exists. Early percentage changes should be interpreted cautiously because small publication counts can magnify relative changes.

**
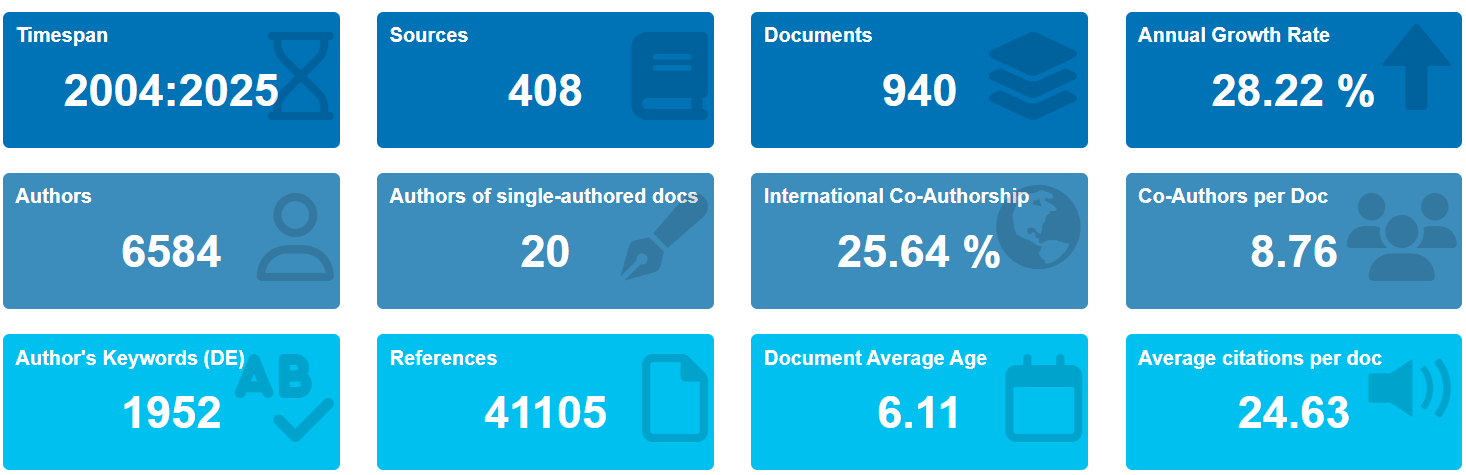
**

**Supplementary Figure 1. General bibliometric characteristics of the included literature.** The panel summarizes the time span, number of sources and documents, annual growth rate, total authors, single-authored documents, international co-authorship rate, co-authors per document, author keywords, references, document age, and average citations per document in the standardized 940-record corpus.

**
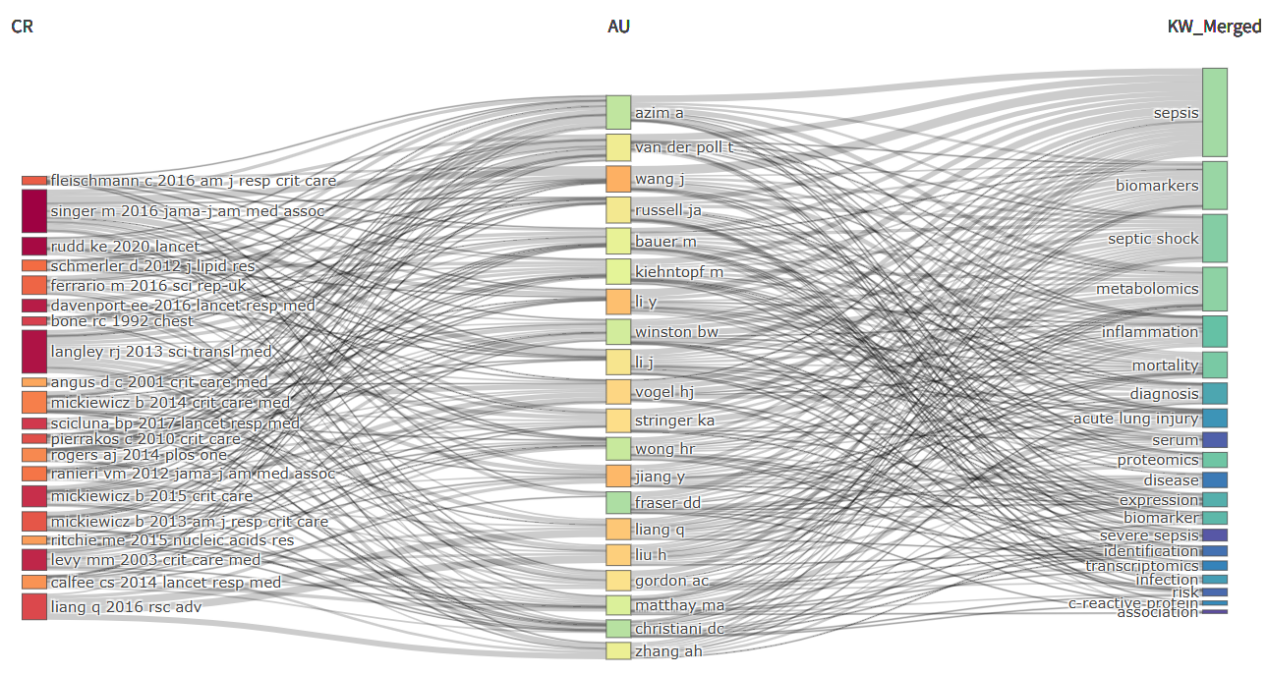
**

**Supplementary Figure 2. Three-field plot of cited references, prolific authors, and high-frequency keywords.** CR denotes cited references, AU denotes authors, and KW_Merged denotes merged keywords after thesaurus-based keyword cleaning. Rectangle height reflects frequency, and gray flow width reflects the number of links between cited references, authors, and keywords. Because this plot is visually dense, it is interpreted as a high-level overview of dominant reference-author-keyword connections rather than as a standalone network of causal relationships.

**
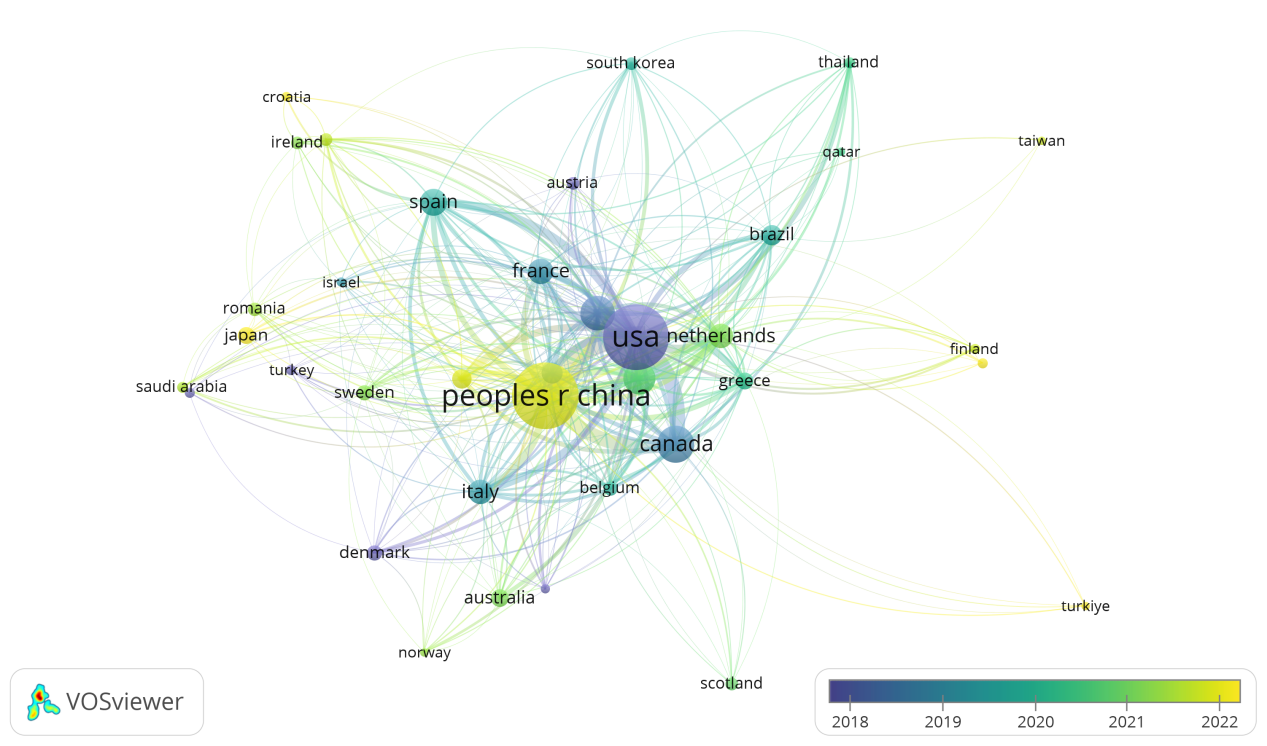
**

**Supplementary Figure 3. Overlay visualization of international collaboration among countries.** In the VOSviewer overlay map, node size represents publication productivity, link width represents collaboration strength, and the color gradient represents the average publication year of each country node, with earlier activity shown in cooler colors and more recent activity shown in warmer colors.

**
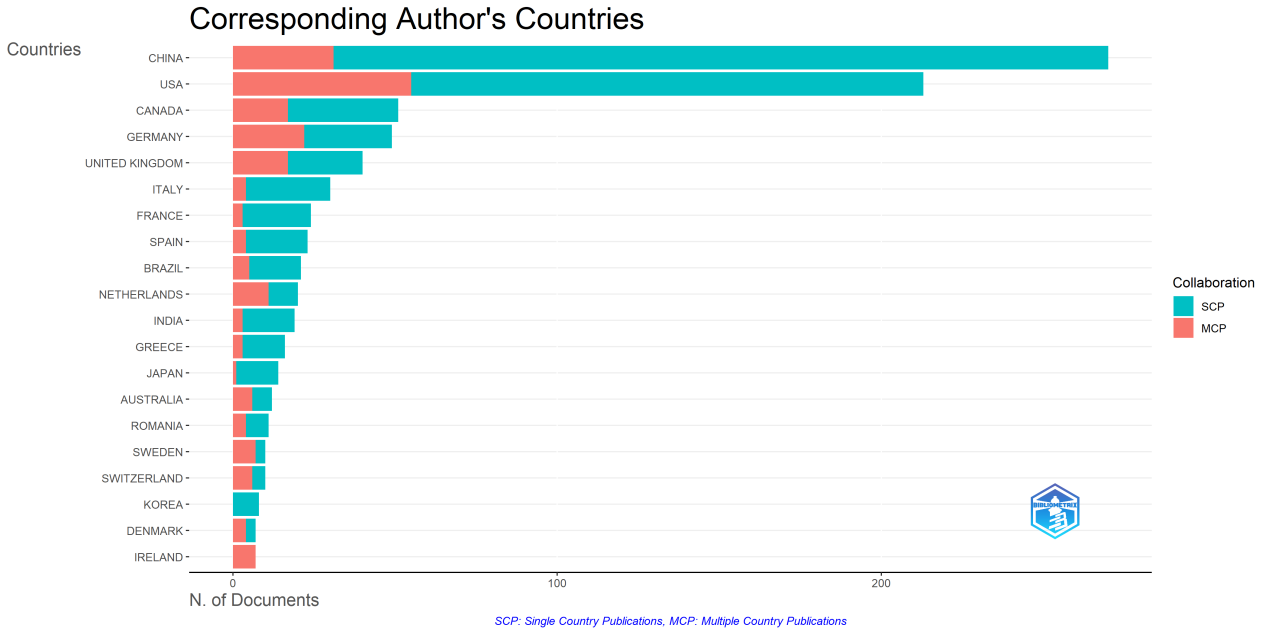
**

**Supplementary Figure 4. Distribution of corresponding authors’ countries and patterns of single-country versus multiple-country publications.** Bars indicate the number of documents led by corresponding authors from each country. SCP denotes single-country publications and MCP denotes multiple-country publications, providing direct support for the corresponding-author analysis in Section 3.2.

**
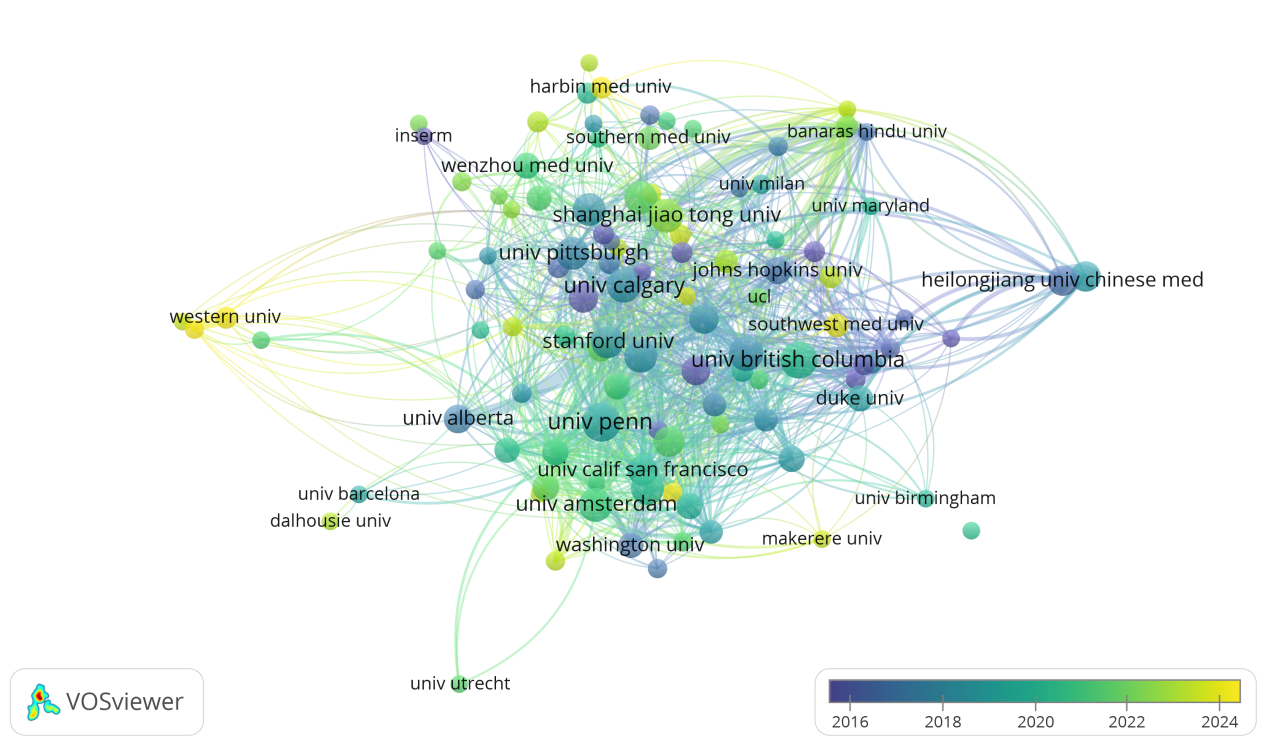
**

**Supplementary Figure 5. Overlay visualization of institutional collaboration network.** Node size reflects institutional productivity, link width reflects collaboration strength, and the color gradient indicates the average publication year of each institutional node.

**
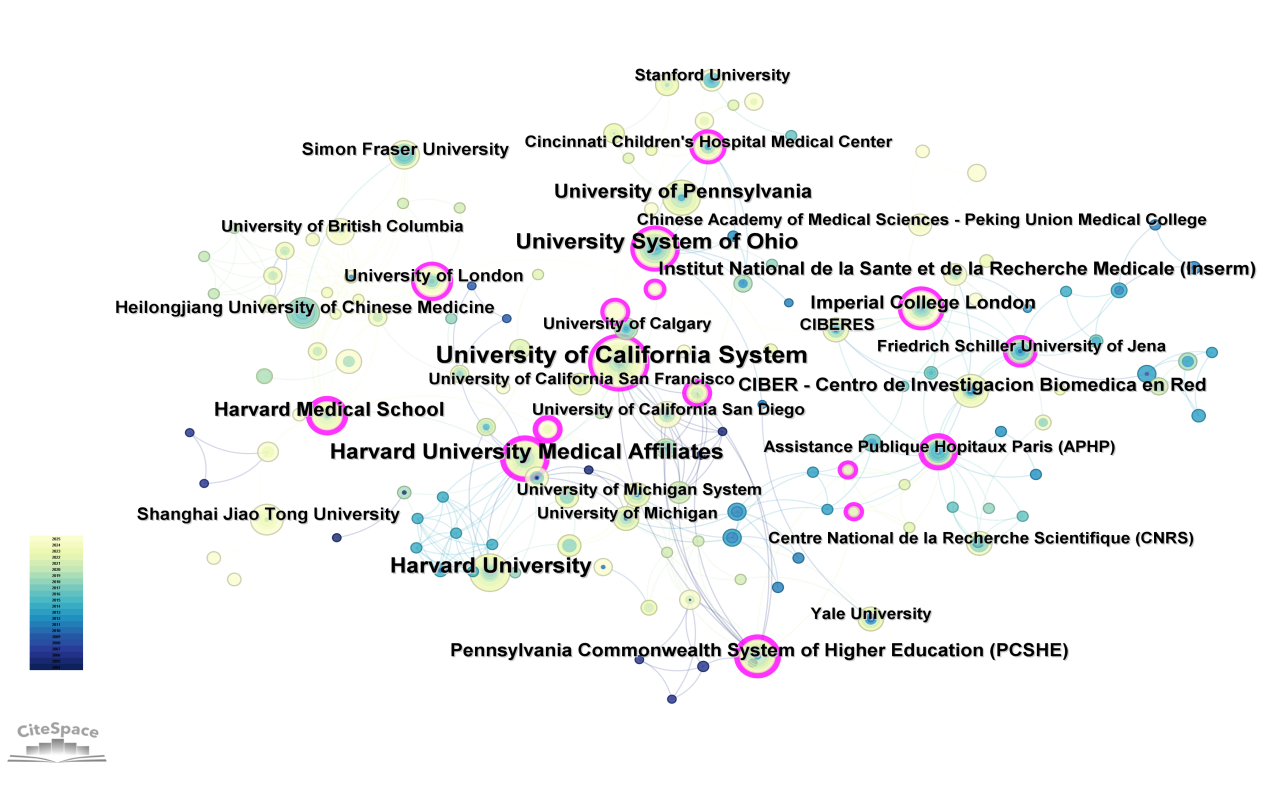
**

**Supplementary Figure 6. Institutional collaboration network in sepsis genetic variation and multi-omics biomarker research.** Each node represents an institution; node size reflects productivity, links represent collaboration, and pink or purple rings in the CiteSpace map indicate institutions with higher betweenness centrality or bridging positions.

**
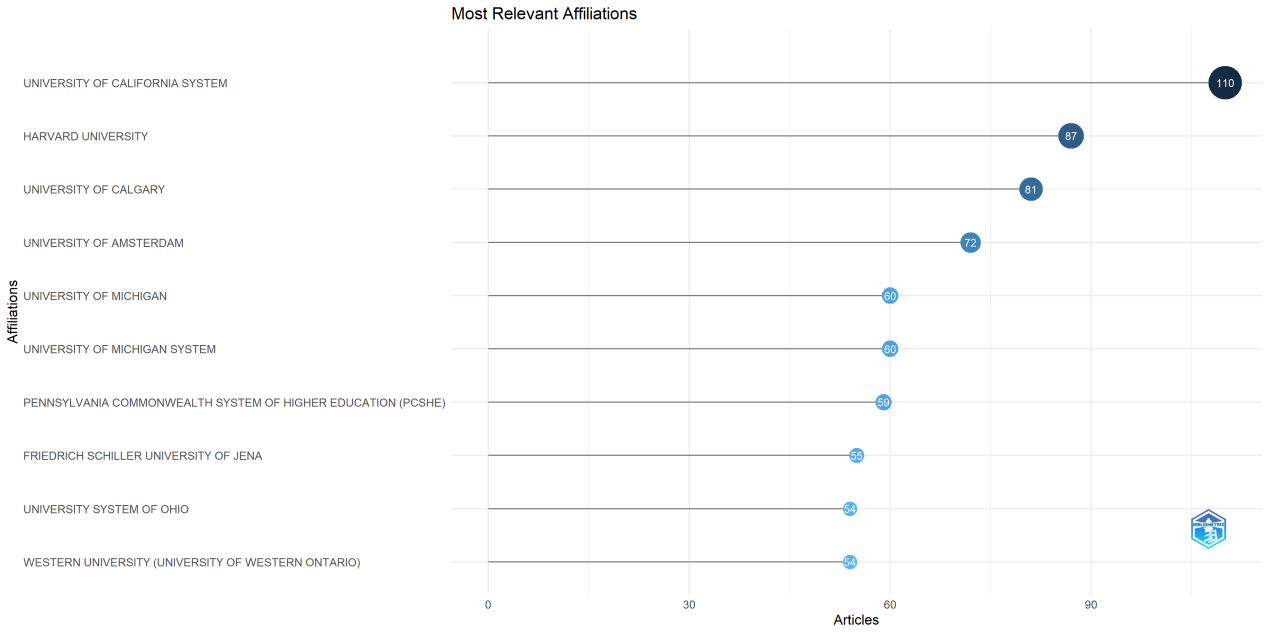
**

**Supplementary Figure 7. Most productive affiliations in sepsis genetic variation and multi-omics biomarker research.** Dot position represents publication count for each affiliation, allowing comparison of institutional productivity in the standardized corpus.

**
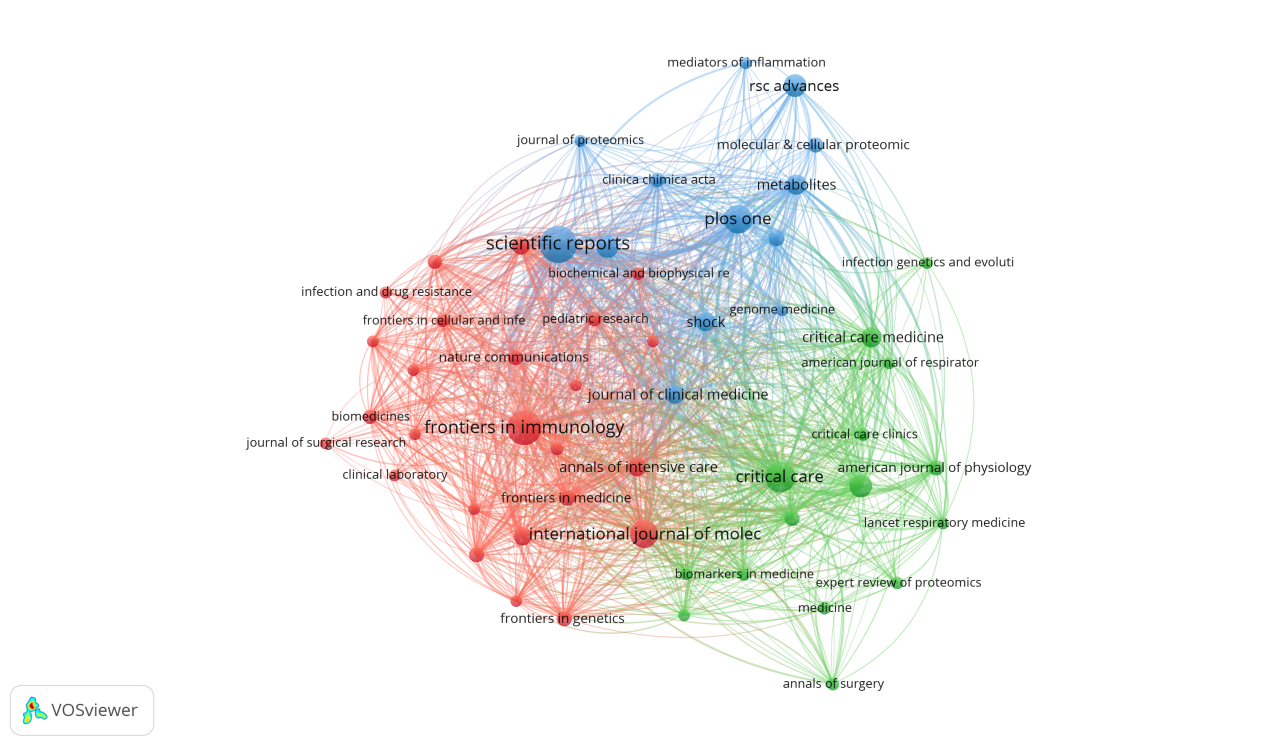
**

**Supplementary Figure 8. Network visualization of core journal sources in sepsis genetic variation and multi-omics biomarker research.** Nodes represent journals, node size indicates source frequency or influence in the mapped network, link width indicates co-occurrence or citation-related connection strength, and cluster colors distinguish groups of journals with similar relational patterns.

**
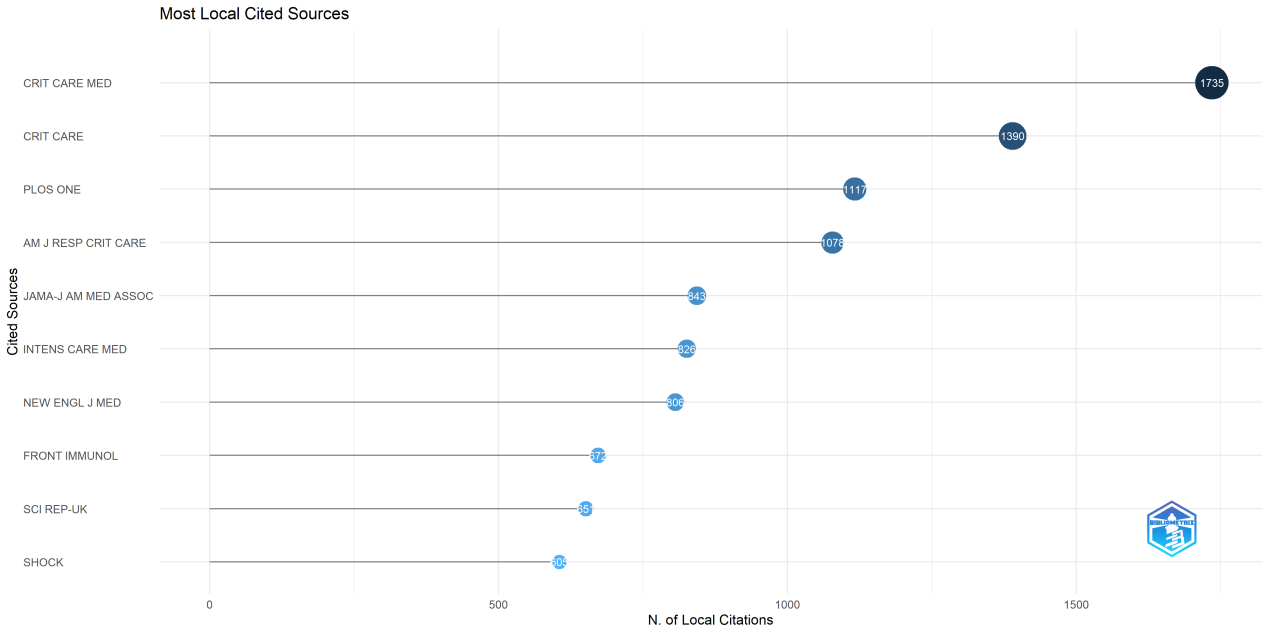
**

**Supplementary Figure 9. Most locally cited journal sources.** Dot position indicates the number of local citations received within the 940-record dataset, emphasizing journals that form the citation backbone of the field.

**
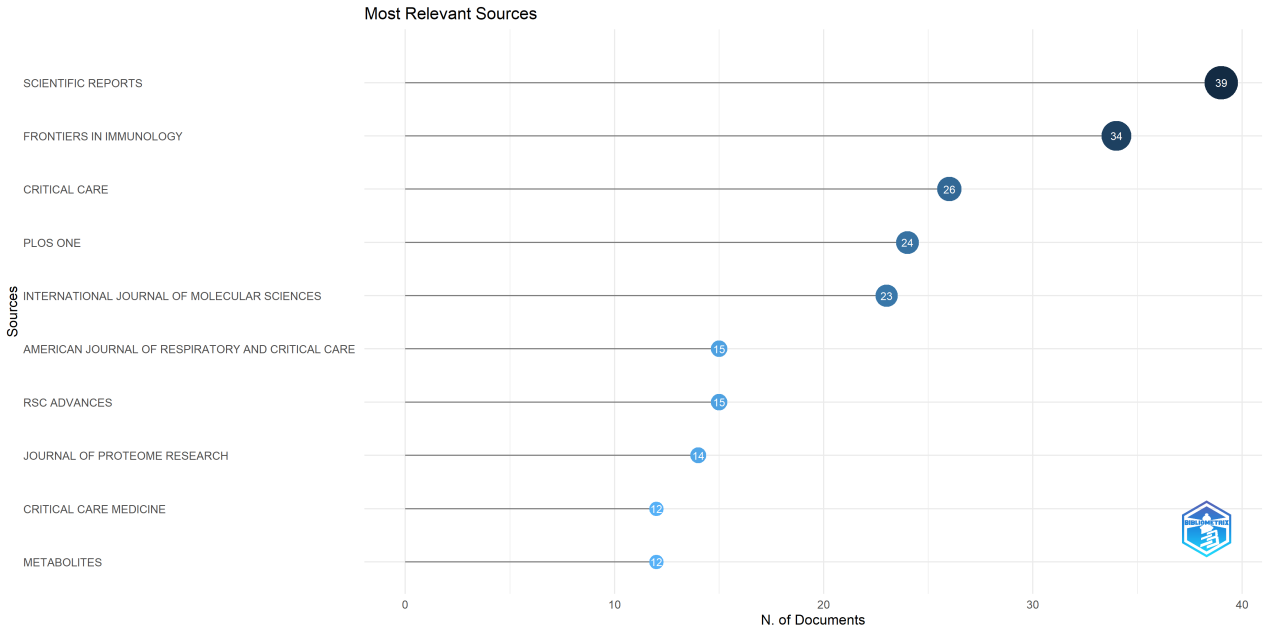
**

**Supplementary Figure 10. Most productive journal sources.** Dot position indicates the number of included articles published by each source, emphasizing publication venues rather than citation influence.

**
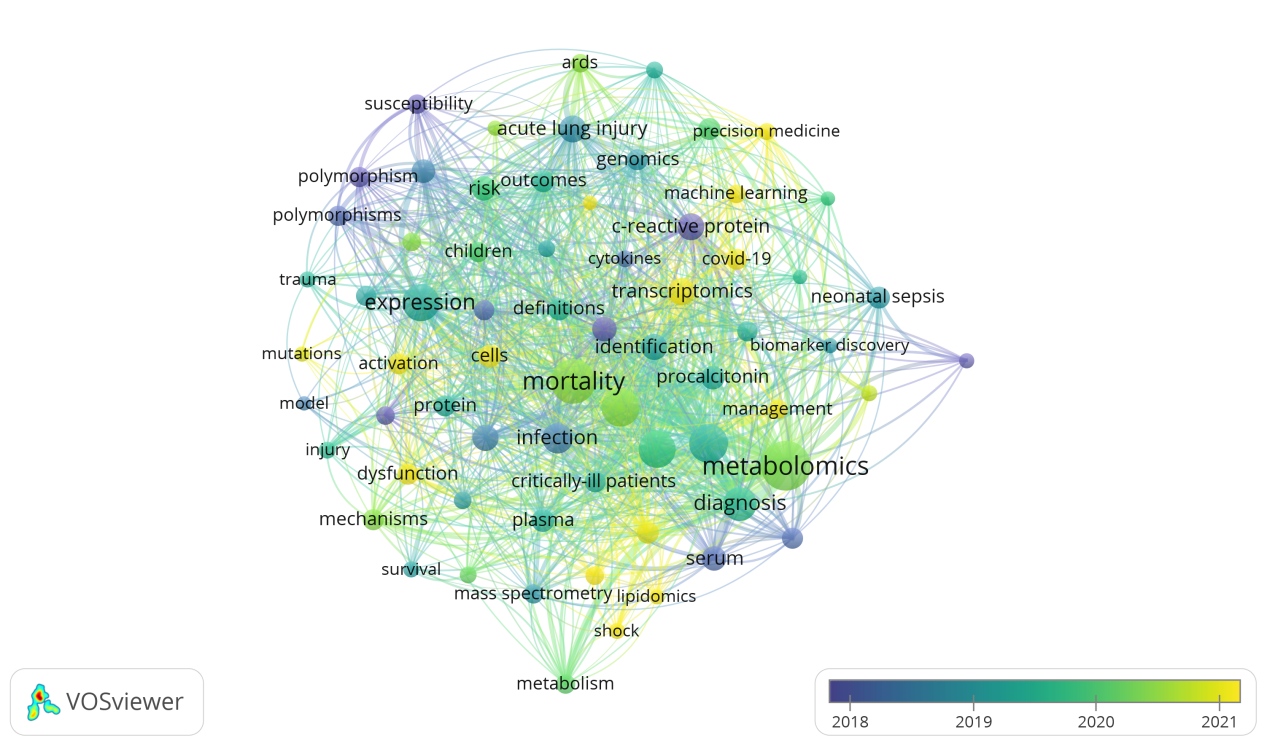
**

**Supplementary Figure 11. Overlay visualization of keyword evolution and recent research emphasis.** Node size reflects keyword frequency, link width reflects co-occurrence strength, and the color gradient represents the average publication year of each keyword. Earlier themes include polymorphism, susceptibility, and conventional inflammatory markers, whereas recent themes include metabolomics, transcriptomics, genomics, precision medicine, machine learning, and lipidomics.

**
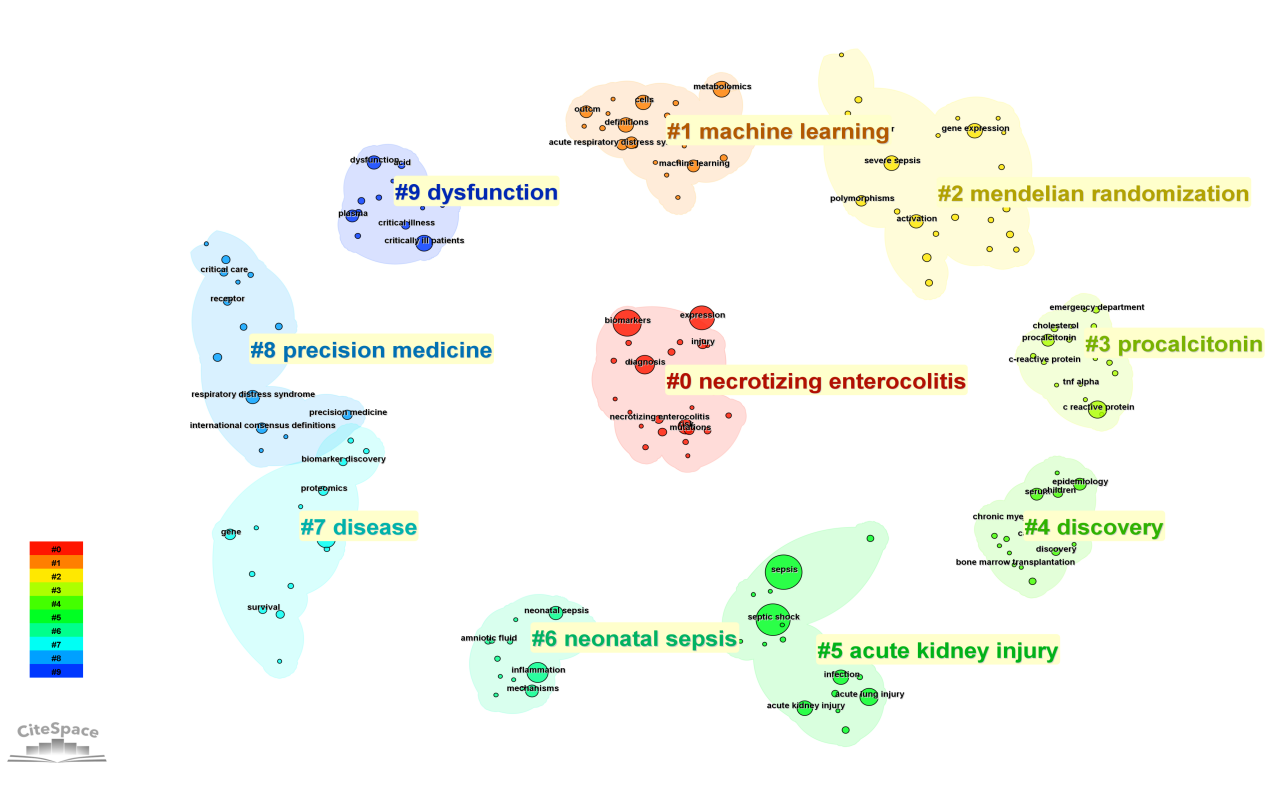
**

**Supplementary Figure 12. Keyword clustering map of major research themes.** Cluster labels summarize co-occurring keyword groups generated by CiteSpace, including clinical-problem clusters such as procalcitonin, acute kidney injury, and neonatal sepsis, and translational-method clusters such as machine learning, Mendelian randomization, and precision medicine.


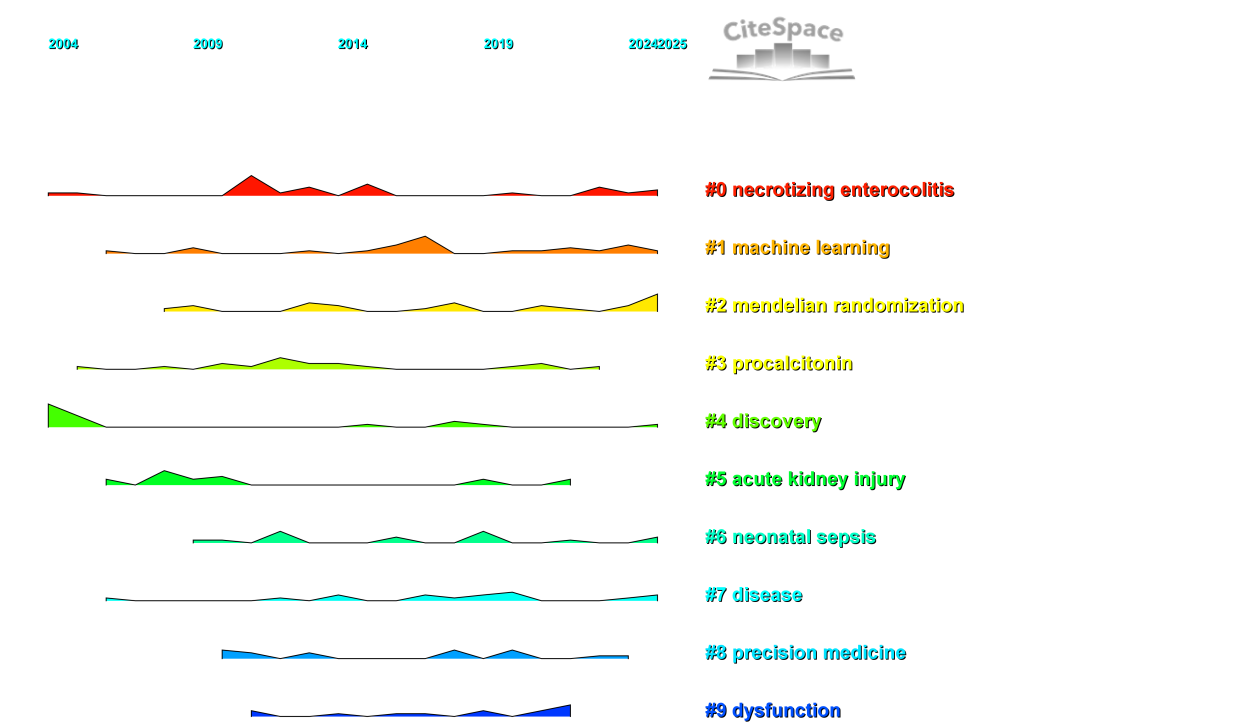


**Supplementary Figure 13. Timeline visualization of keyword clusters and thematic evolution.** The timeline displays the temporal persistence and emergence of keyword clusters, showing progression from earlier discovery-oriented and clinical-context themes toward more recent machine learning, Mendelian randomization, precision medicine, and dysfunction-related topics.
